# A Self-Powered Density-Based Device for Automatic Mixed-Oil Cutting in Field Pipelines

**DOI:** 10.3390/s25103030

**Published:** 2025-05-11

**Authors:** Zhen Zhang, Yonggang Zuo, Huishu Liu, Biao He

**Affiliations:** 1 Army Logistics Academy of PLA, Chongqing 401331, China; 13883017576@139.com (Z.Z.); zyg938@163.com (Y.Z.); 2 School of Petroleum Engineering, Chongqing University of Science and Technology, Chongqing 401331, China; hsliu0820@163.com

**Keywords:** densitometer, float sensor, self-powered, modular design, real-time monitoring, mixed-oil cutting, field oil pipeline, instrumentation, sensor

## Abstract

Efficient oil transportation in field-deployed mobile pipelines is critical, but mixed-oil zones at interfaces reduce quality and increase waste, necessitating effective interface detection and cutting. Existing online densitometers, such as vibrating tube or high-accuracy magnetic suspension types, typically require external power, limiting their use in remote or emergency/temporary field operations. A self-powered device is presented that leverages gravitational force variations acting on a float to detect density changes and trigger automatic cutting. Validated with gasoline, diesel, kerosene, and water, it achieves a 10 kg/$m^{3}$ resolution, deemed sufficient for functional batch separation in its target application, with switching times of 61–395 s for density differences (760–835 kg/$m^{3}$). It supports 20–90% blending ratios, with a vent mitigating gas effects. The modular, robust, self-powered design suits emergency operations, offering a practical alternative to powered systems. Future work targets improved resolution and environmental testing.

## 1. Introduction

The efficient transportation of oil via pipelines is critical for meeting global energy demands [1]. In field-deployed mobile pipelines, used in emergency scenarios (e.g., disaster response, military logistics) or temporary operations (e.g., remote oilfields) [2,3] sequential transportation—where different oil products are conveyed consecutively within the same pipeline—maximizes operational efficiency [1,4]. However, this method results in a mixed-oil zone at the interface between products, which can degrade product quality, increase waste, and pose safety concerns [5]. Effective mixed-oil detection and cutting is essential to minimize product loss and environmental impact, supporting sustainable oil transportation [6].

Current online density measurement methods, such as precise vibrating tube [7] or radiation-based densitometers [8], are widely used in permanent installations. Highly accurate density measurements are also possible with specialized equipment like magnetic suspension balances [8,9]. However, these instruments often require significant external power, stable laboratory conditions, or complex infrastructure, making them less practical for remote, rapidly deployed mobile settings where power is unreliable and robustness is paramount [7,8]. While advanced interface detection sensors are being developed [10] they often face similar power and portability constraints in mobile contexts.

Beyond operational efficiency, managing mixed-oil formation aligns with global sustainability goals by reducing contamination risks [11,12]. Various other density measurement techniques (e.g., ultrasonic [13,14], differential pressure [15]) exist, but typically require external power and signal processing. Traditional hydrometers, while simple, are inherently unsuitable for automated, inline monitoring in dynamic pipeline flows due to their operating principle requiring manual reading or static conditions. The trend towards self-powered sensors, often based on Triboelectric Nanogenerators (TENGs) [16,17,18], is promising for remote applications, but these currently focus on parameters like fluid velocity or energy harvesting, lacking the specific precision or mechanism required for density-based mixed-oil cutting.

**Figure 1:**
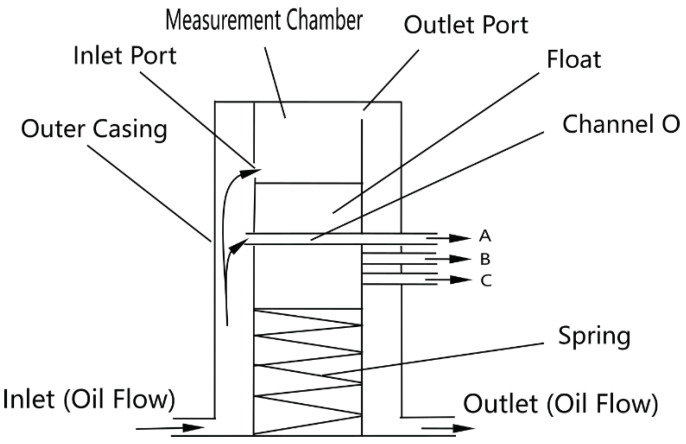
Schematic diagram of the density setting and adjustment device. A, B, C are the signal pipes cooresponding to different density.

This paper presents a novel self-powered, density-based automatic cutting device designed specifically for field-deployed mobile pipelines. It prioritizes robustness, simplicity, and power independence over the ultra-high precision demanded in permanent pipeline systems. The device integrates density sensing (via float displacement driven by gravitational/buoyancy forces) with mechanical actuation to enable automatic mixed-oil cutting, offering a practical solution for challenging field environments, as illustrated in the schematic design (Figure 1).

## 2. Materials and Methods

### 2.1. Device Composition

**Figure 2:**
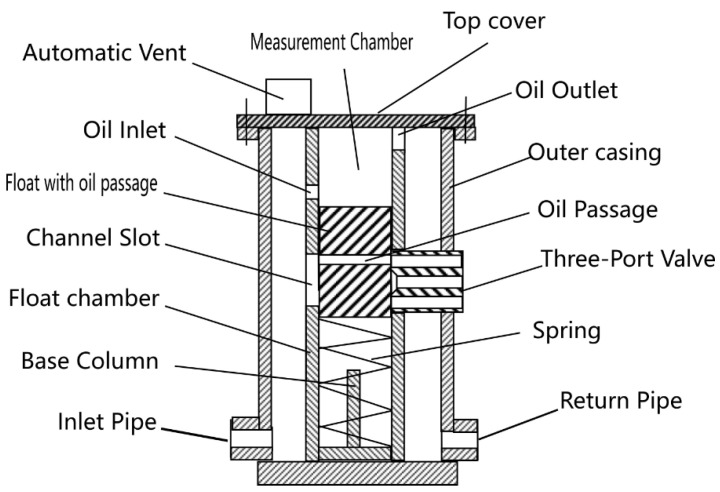
Exploded view of the device, showing component arrangement.

The device comprises a top cover, outer casing, float with oil passage, float chamber, spring, three-port valve, and automatic vent, as depicted in Figure 2.

### 2.2. Key Component Design

- **Top Cover**: Includes an automatic vent to prevent gas interference, bolted for maintenance.
- **Float Chamber**: Contains the float, spring, and base column, with oil inlet/outlet and adjustment rod slots.
- **Three-Port Valve**: Controls cutting signals with channels for gasoline, mixed oil, and diesel.
- **Outer Casing**: Houses inlet/return pipes and secures the float chamber.

### 2.3. Modular Design

**Figure 3:**
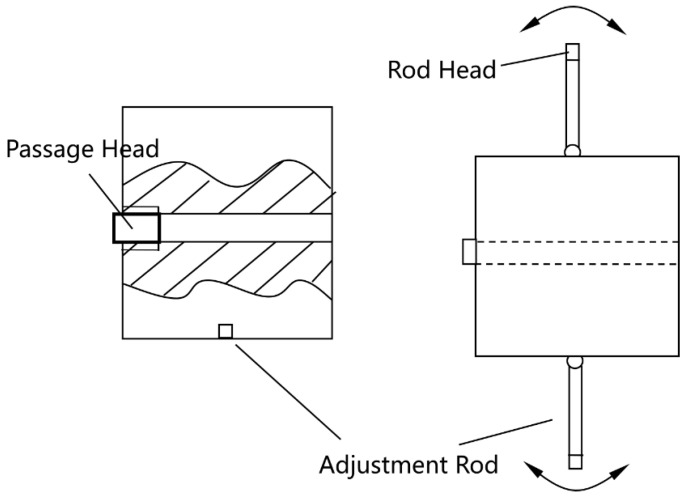
Schematic of the float design.

**Figure 4:**
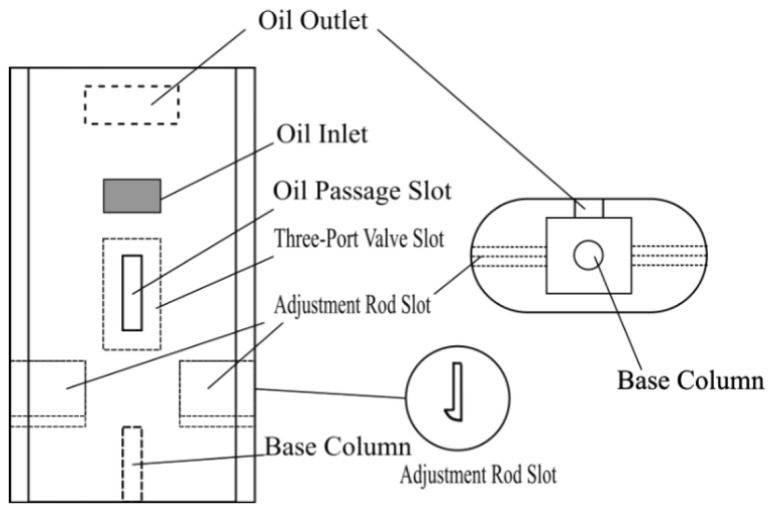
Schematic of the float chamber.

The float and float chamber are modular, allowing interchangeability to accommodate various fluid density ranges (e.g., gasoline, diesel, kerosene, or heavier oils), enhancing versatility. This design enables adaptation to different fluids by swapping components, as shown in Figure 3 and Figure 4, minimizing the need for structural redesign.

### 2.4. Detailed Design

**Figure 5:**
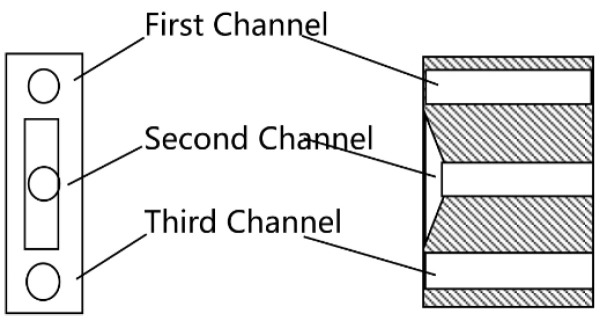
Schematic of the three-port valve.

**Figure 6:**
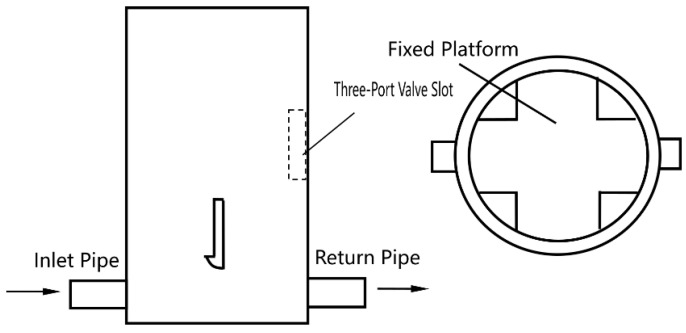
Schematic of the outer casing.

Detailed schematics of individual components are provided for clarity: the float (Figure 3), float chamber (Figure 4), three-port valve (Figure 5), and outer casing (Figure 6).

### 2.5. Material Selection

The float is constructed from stainless steel (316L, Huaxiao Metal, Shanghai, China), offering high corrosion resistance to oil and environmental exposure, with a pitting resistance equivalent number (PREN) > 25, ensuring durability in harsh field conditions. The spring is made from a nickel-chromium alloy (Inconel X-750, HWZ, Wuxi, China), selected for its fatigue life (>$10^{6}$ cycles) and stability under repeated compression. The outer casing uses an aluminum alloy (6061-T6, Xingfa Aluminium, Foshan, China) with an anodized coating to resist corrosion while maintaining lightweight portability (density 2.7 g/${\mathrm{cm}}^{3}$), critical for mobile pipelines, as illustrated in Figure 2, Figure 3, Figure 4, Figure 5 and Figure 6.

### 2.6. Working Principle

The device aims to automatically detect and cut the mixed-oil interface during sequential oil transportation in field mobile pipelines. It employs a purely mechanical design driven by gravitational force variations acting on a float, ensuring self-powered operation without external energy input. The core function is to monitor oil density continuously in dynamic flow conditions and, based on pre-set thresholds corresponding to float displacement, output control signals to switch valves, diverting oil products to separate lines. The device requires continuous flow to operates, as it relies on fluid interaction with the float, and is not designed for static conditions, as shown in Figure 1.

**Figure 7:**
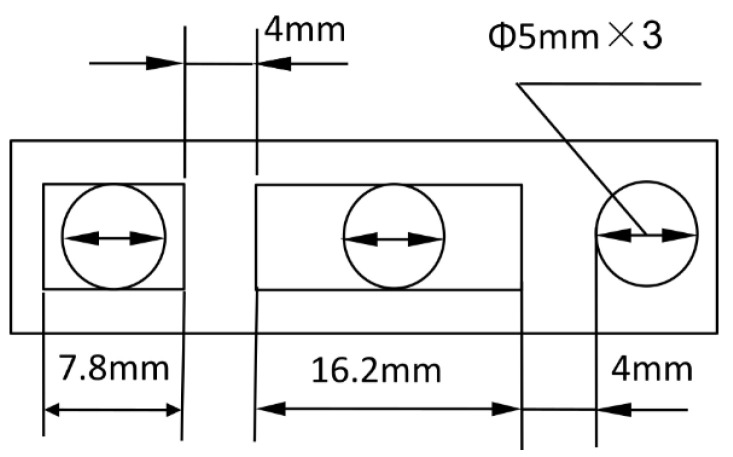
Dimensions of the three-port valve.

The device operates on buoyancy changes caused by oil density variations. Density changes affect the weight of oil in a measurement chamber, compressing a spring per Hooke’s Law, which indirectly measures density through float displacement. Figure 1 illustrates the working principle: oil enters through an inlet pipe, with a portion flowing through channel O to a cutting device, while the rest enters the measurement chamber. As mixed oil increases density, the float moves downward, aligning channel O with different pipes (A, B, C) to signal valve switches for gasoline, mixed oil, or diesel. The dimensions are shown in Figure 7.

### 2.7. Experimental Setup

**Figure 8:**
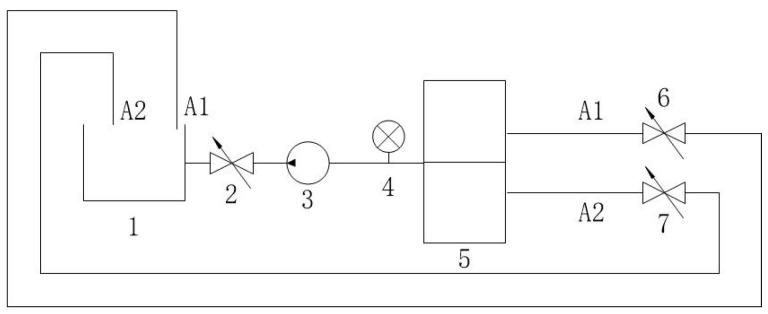
Test bench schematic for density-controlled pilot valve on-off test. 1—Oil Storage Container; 2, 6, 7—Ball Valves; 3—Oil Pump; 4—Pressure Gauge 5—Density Control Pilot Valve; A1—Light Oil Passage; A2—Heavy Oil Passage.

**Figure 9:**
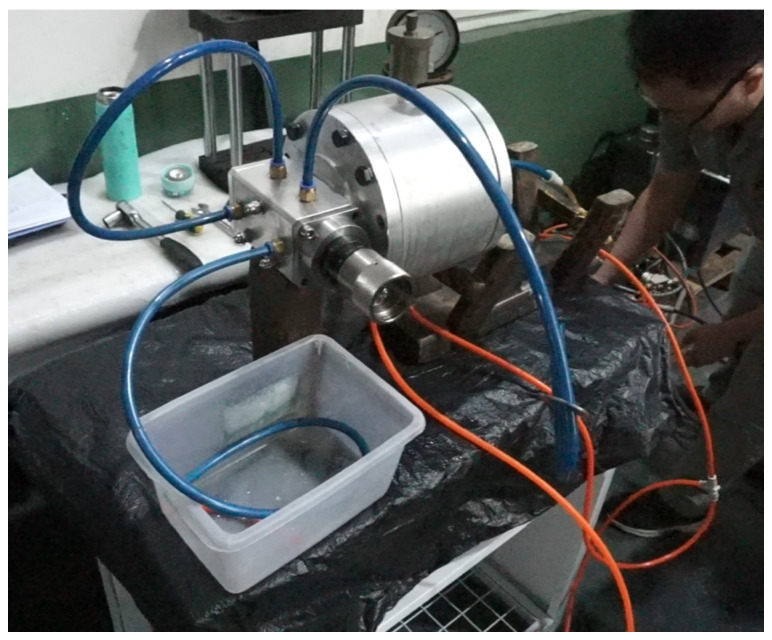
Photograph of the density control pilot valve test bench.

**Figure 10:**
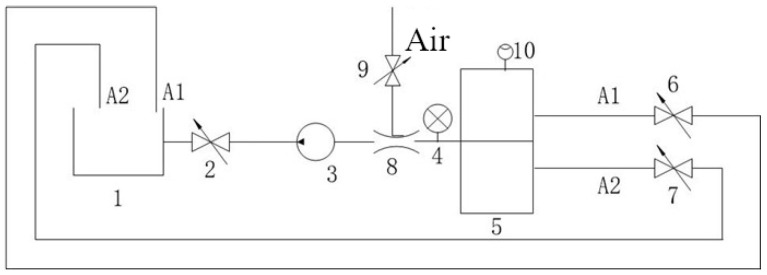
Schematic diagram of the gas test system. 1—Oil Storage Container; 2, 6, 7, 9—Ball Valves; 3—Oil Pump; 4—Pressure Gauge; 5—Density Control Pilot Valve; 8—Venturi Tube; 10—Automatic Venting Device; A1—Light Oil Passage; A2—Heavy Oil Passage.

**Figure 11:**
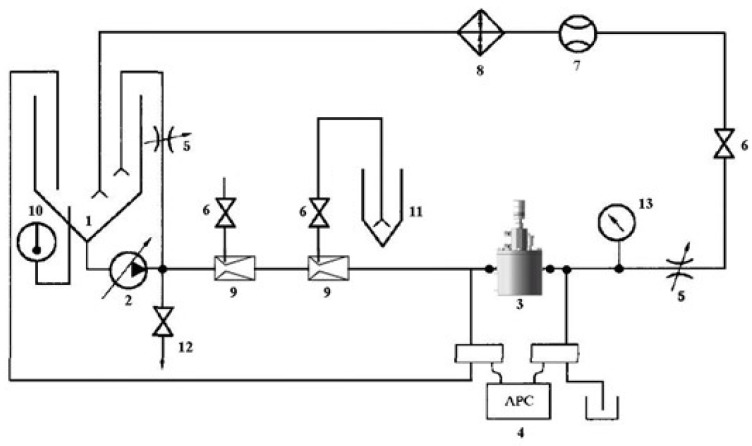
Mixed-oil test system schematic. 1—Oil Tank; 2—Oil Pump; 3—Tested Pilot Valve; 4—Particle Counting System; 5—Control Valve; 6—Valve; 7—Flowmeter; 8—Heat Exchanger; 9—Venturi; 10—Thermometer; 11—Waste Oil Tank; 12—Sampling Valve; 13—Pressure Gauge.

Experiments validated the device using a test bench (Figure 8 and Figure 9), assessing density detection, switching times, gas effects (Figure 10), and mixed-oil cutting (Figure 11). Test media included gasoline (760 kg/$m^{3}$), diesel (835 kg/$m^{3}$), kerosene (798 kg/$m^{3}$), and water (998 kg/$m^{3}$).

## 3. Results

**Figure 12:**
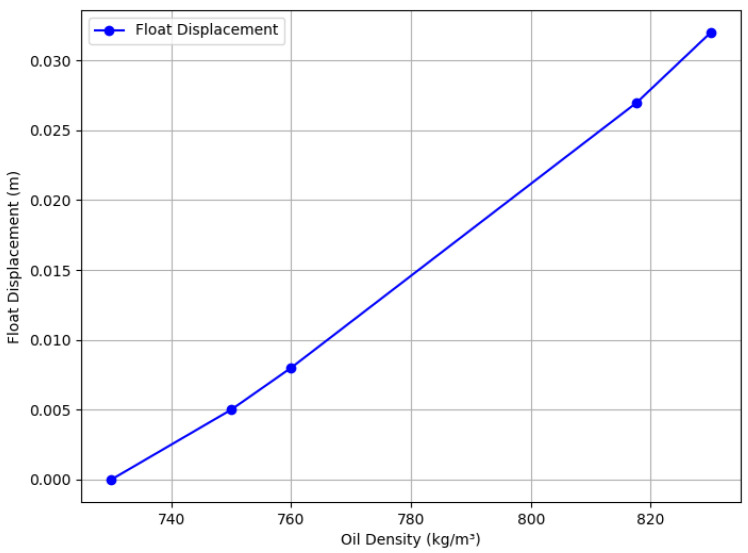
Plot of float displacement vs. oil density, illustrating the relationship derived from Table 1.

**Figure 13:**
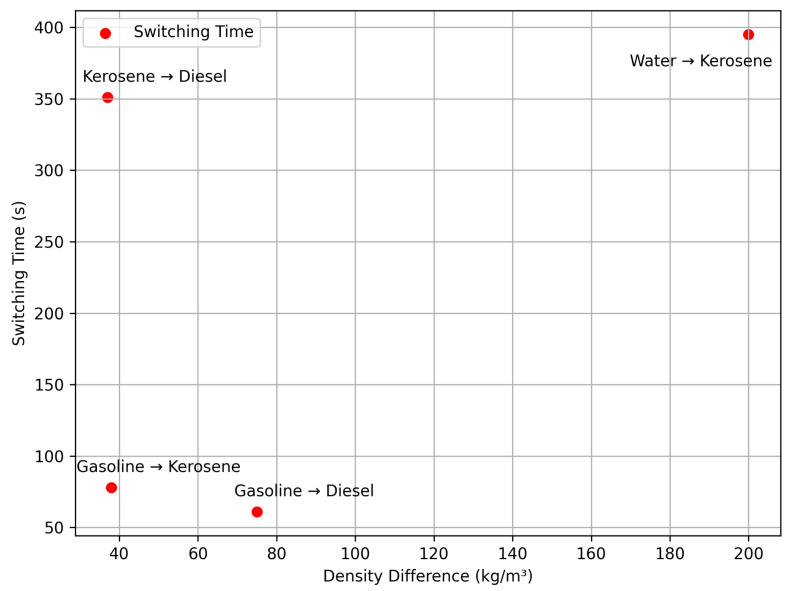
Plot of switching time vs. density difference, showing the relationship from Table 4.

**Figure 14:**
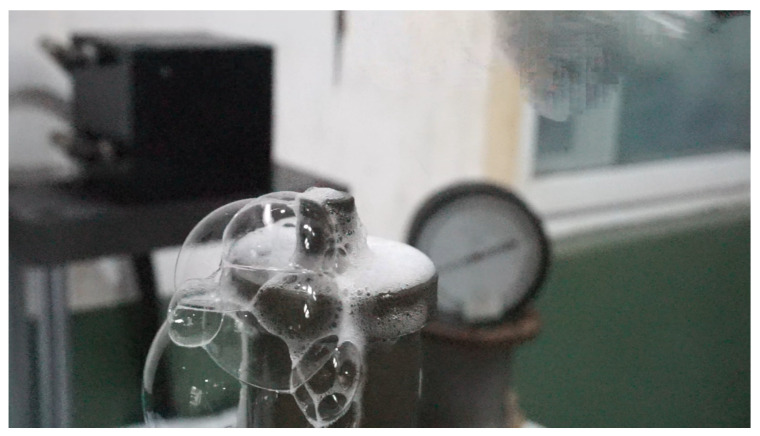
Photograph of the automatic venting device during operation, showing bubble formation.

Building on the design, this section presents theoretical and experimental results, with performance summarized in Table 1, Table 2, Table 3, Table 4, Table 5 and Table 6 and visualized in Figure 12, Figure 13 and Figure 14.

### 3.1. Relationship Between Oil Density and Float Position

Assuming gasoline (730 kg/$m^{3}$) followed by diesel (830 kg/$m^{3}$), with a measurement chamber area of 0.01 $m^{2}$, initial liquid level of 0.1 m, and maximum of 0.132 m, the spring compression changes by 0.032 m. The float weight is 1 N. Forces are calculated as:

(1) $$F_{\min}=\rho_{\mathrm{gasoline}}\cdot g\cdot V_{\min}+G_{\mathrm{float}}=8.16\;N$$

(2) $$F_{\max}=\rho_{\mathrm{diesel}}\cdot g\cdot V_{\max}+G_{\mathrm{float}}=11.78\;N$$

The spring constant k=112N/m, with initial compression of 0.0728 m. The relationship between density (ρ) and float displacement (*x*) is:

(3) $$x=\frac{\rho \cdot g\cdot S\cdot h_{\mathrm{initial}}+G_{\mathrm{float}}-k\cdot B}{k-\rho \cdot g\cdot S}$$

At 750 kg/$m^{3}$, the gasoline:diesel ratio is 8:2; at 817.7 kg/$m^{3}$, it is 1.23:8.77. Results are visualized in Figure 12.

### 3.2. Density-Controlled Pilot Valve On-Off Test

A test bench (Figure 8) validated density identification using media listed in Table 2. Results (Table 3) show a resolution of 10 kg/$m^{3}$, with A1 open when density is below the set point and A2 open when above. The physical setup is shown in Figure 9.

### 3.3. Two Test Media Test

Switching times between media (Table 4) confirm automatic cut-off, with times related to density differences, visualized in Figure 13.

### 3.4. Gas Effect

The automatic vent effectively removes gas, maintaining accuracy, as shown in Table 5 and Figure 10, Figure 11, Figure 12, Figure 13 and Figure 14. Over prolonged use, residual gas accumulation could slightly affect float responsiveness, but the vent design minimizes this risk, as evidenced by bubble release in Figure 14.

### 3.5. Mixed-Oil Experiment

Simulating field conditions in mobile pipelines, experiments used gasoline (730 kg/$m^{3}$) and diesel (830 kg/$m^{3}$), with the setup shown in Figure 11. At a threshold of 750 kg/$m^{3}$ (80% gasoline, 20% diesel), A1 closes and A2 opens when diesel reaches 20%, achieving automatic cutting. At 817 kg/$m^{3}$ (10% gasoline, 90% diesel), similar results occur. These thresholds were selected based on typical operational ranges for mobile pipelines in emergency scenarios, where broader blending ratios (20–90%) are acceptable to prioritize rapid deployment.

## 4. Discussion

The experimental results demonstrate that the proposed self-powered device measures fluid density with a resolution of 10 kg/$m^{3}$, enabling functional detection and cutting of mixed-oil interfaces in field-deployed mobile pipelines, as validated by Table 2, Table 3 and Table 4 and Figure 8, Figure 9, Figure 10, Figure 11, Figure 12, Figure 13 and Figure 14. The purely mechanical design translates density variations into valve actuation via float displacement, ensuring robustness and self-powered operation for field applications.

### 4.1. Operational Considerations

Compared to the high standards required for permanent commercial pipeline systems, where optimizing product yield necessitates cutting points below 7% blending ratios and requires densitometer resolutions much finer than 1 kg/$m^{3}$ (e.g., vibrating tube types [7] or specialized magnetic suspension densitometers [8]), the proposed device’s 10 kg/$m^{3}$ resolution (Table 1, Figure 12) is less precise. However, this resolution is tailored for and deemed sufficient for its intended niche: emergency or temporary mobile pipelines. In these specific contexts, operational priorities shift. Self-powered operation, robustness, portability, rapid deployment, and simplicity often outweigh the need for ultra-high accuracy [1,2,3]. External power is frequently unavailable [7,8], making conventional high-precision, powered densitometers [7,8,14,19] impractical. The primary goal in such field scenarios is often rapid, automated, functional separation of different bulk product batches (e.g., distinguishing gasoline from diesel) within broader acceptable interface cuts (like the 20–90% range tested), rather than the fine optimization required in high-value commercial operations. Our device provides a practical, autonomous solution for this specific need (Table 6). Future improvements, such as finer spring calibration or optimized float design, could potentially reduce the resolution toward 5 kg/$m^{3}$ if required for slightly more demanding field applications.

In field conditions, factors beyond resolution affect performance. Issues like air bubbles from oil mixing or solid particle settlement (e.g., sand, debris) could affect float response (Table 5, Figure 10, Figure 11, Figure 12, Figure 13 and Figure 14). Air bubbles may reduce effective density, causing float misplacement by 0.002–0.005 m for 1–2% gas content, although this is mitigated by the automatic vent (Figure 1). Solid particles could settle, impeding float movement; however, the modular design (Figure 3 and Figure 4) allows for field cleaning, and future iterations may incorporate inlet filters. The device’s reliance on continuous flow (Figure 1) limits its use in static conditions, unlike some other sensor types, but a bypass flow inducer could potentially address this in future designs for specific intermittent flow scenarios. These challenges will be studied further in extended field trials, building on setups in Figure 8, Figure 11, Figure 12, Figure 13 and Figure 14.

### 4.2. Environmental Influences

Temperature and viscosity variations, common in field operations [4,11], can influence performance. Temperature affects oil density ($\rho =\rho_{0}\left[1-\beta \left(T-T_{0}\right)\right]$, where $\beta \approx 0.0007\;K^{-1}$), potentially shifting float displacement by 0.001 m per 10 °C change for gasoline (Figure 12), corresponding to an error of approximately 3 kg/$m^{3}$. Viscosity introduces a drag force ($F_{d}=6\pi \eta rv$). For typical parameters (e.g., 0.6 mPa·s for gasoline, float radius r≈0.05 m, velocity v≈0.01 m/s), $F_{d}\approx 0.005\;N$, which is minor compared to the buoyancy force (8–12 N). Turbulence is minimized by the chamber’s design (Figure 4). While these effects were not specifically isolated and tested across wide ranges in our controlled lab setup, the 10 kg/$m^{3}$ resolution inherently accounts for some level of operational variability. Future field trials will assess performance across typical operational temperatures (e.g., 0–50 °C) and viscosities (0.5–5 mPa·s).

Scalability to Larger Pipelines. Switching times (61–395 s, Table 4, Figure 13) depend on flow rate and chamber volume. For larger pipelines, increased flow rates could reduce response times proportionally (t∝1/Q), but larger chambers may counteract this. Scaling requires optimizing chamber size (Figure 4) and valve speed (Figure 5), potentially achieving sub-minute responses.

Temperature and Pressure Impacts. Experiments did not evaluate temperature or pressure effects. Future trials will test 0–50 °C and up to 5 bar to quantify impacts on accuracy and valve performance (Figure 8, Figure 11, Figure 12, Figure 13 and Figure 14).

### 4.3. Fluid Compatibility

While optimized for gasoline and diesel (Table 1), the modular design supports other fluids (e.g., kerosene at 790 kg/$m^{3}$, heavy oils up to 950 kg/$m^{3}$) by adjusting the float and spring (Figure 3 and Figure 4). Minor calibration may be needed for extreme density ranges, as tested with kerosene and water (Table 4), with future tests planned for broader fluids, leveraging setups in Figure 11 and Figure 14.

Limitations include the current resolution constraint for applications requiring high precision, the need for further testing under wider environmental conditions (temperature, pressure, vibration), and the reliance on continuous flow for operation. Future work will explore resolution enhancement, quantify environmental robustness through field trials, investigate static operation possibilities, and consider integration with low-power telemetry for remote monitoring.

## 5. Conclusions

This paper presents a novel self-powered, density-based automatic cutting device for mixed-oil interfaces in field-deployed mobile pipelines. It achieves a resolution of 10 kg/$m^{3}$, demonstrated to be sufficient for reliably detecting and cutting interfaces between common products like gasoline (730 kg/$m^{3}$) and diesel (830 kg/$m^{3}$) within typical field-operational blending ranges (20–90%), which is suitable for functional separation in many temporary or emergency field scenarios. Its key advantages lie in its self-powered operation, eliminating the need for external power in remote or emergency settings, and its modular, robust mechanical design, allowing adaptation to various fluids and ensuring portability and simplicity. This makes it highly suitable for its intended niche of emergency or temporary operations (e.g., disaster response, military logistics, remote sites) where conventional high-precision, powered densitometers [7,8,19] are impractical. While less precise than permanent pipeline instruments, it excels in power-scarce, demanding field environments, reducing waste and enhancing operational efficiency, as validated by experiments. Future work will focus on extended field trials to assess long-term reliability, quantify performance under varying environmental conditions (temperature, pressure), improve resolution, evaluate compatibility with a wider range of field fluids, and explore options for static operation and remote monitoring.

## 6. Patents

Zuo, Y.; Zhang, Z. etc. “Self-Powered Mixed-oil Cutting Device” Chinese Patent CN [CN113048268A], [2021-06-29].

## Figures and Tables

**Table 1:** Relationship Between Oil Density and Float Position.

Float Displacement (m)	Density (kg/$m^{3}$)
0	730
0.005	750
0.008	760
0.027	817.7
0.032	830

**Table 2:** Test Media Properties.

Medium	Density (kg/$m^{3}$)
Gasoline	760
Kerosene	798
Diesel	835
Water	998

**Table 3:** Density-Controlled Pilot Valve Results.

Set Point (kg/$m^{3}$)	Valve State	Measured Density (kg/$m^{3}$)
770	A1 Open	760
770	A2 Open	835

**Table 4:** Density Control Pilot Valve On-Off Test with Two Test Media.

No.	Medium X	X Density (kg/$m^{3}$)	Medium Y	Y Density (kg/$m^{3}$)	Set Point (kg/$m^{3}$)	Switching Time (s)
1	Gasoline	760	Diesel	835	770	61
2	Gasoline	760	Kerosene	798	770	78
3	Kerosene	798	Diesel	835	820	351
4	Water	998	Kerosene	798	810	395

**Table 5:** Gas Effect on Measurement Accuracy.

Gas Content (%)	Density Error (kg/$m^{3}$)
0	0
1	2
2	5

**Table 6:** Comparison of Density Measurement Devices.

Feature	Proposed Device	Vibrating Tube Densitometer [7]	Radiation-Based Densitometer [8]
Power Requirement	None (Self-Powered)	External Power	External Power
Resolution (kg/$m^{3}$)	10	0.1	1–5
Approximate Cost	Low	High	High
Field Suitability ^a^	High (Mobile, Robust, Self-Powered)	Low (Power-Dependent, Infrastructure)	Moderate (Safety Concerns, Infrastructure)

^a^ Field suitability assessed based on the intended application in remote, mobile, power-scarce field contexts. Requirements differ significantly for permanent pipeline optimization.

## Data Availability

The original contributions presented in this study are included in the article material. Further inquiries can be directed to the corresponding author.
